# Special Issue “Metal–Organic Coordination Compounds: Synthesis, Structure, Spectra, Applications”

**DOI:** 10.3390/ijms27062824

**Published:** 2026-03-20

**Authors:** Georgiy V. Girichev, Nina I. Giricheva

**Affiliations:** 1 Department of Physics, Ivanovo State University of Chemistry and Technology, Sheremetevsky Avenue 7, Ivanovo 153000, Russia; 2 Nanomaterial Research Institute, Ivanovo State University, Ermaka Str. 39, Ivanovo 153025, Russia; n.i.giricheva@mail.ru

## 1. Introduction

Compounds of metals with organic moieties cover the widest classes of chemical substances, with various applications in the chemical industry, catalysis, microelectronics, sensors, medicine, pharmacology, etc. Coordination chemistry has evolved into an extremely broad field, encompassing classical coordination complexes, organometallic compounds, and branched organometallic structures such as metal–organic frameworks (MOFs).

This set of chemical compounds, whose general name is indicated in the title of this Special Issue, “Metal–Organic Coordination Compounds”, represents a type of “border zone” between two classical types of chemical compounds—organic and inorganic. Therefore, they often possess a number of unique properties not found in either inorganic or organic substances.

In the literature, two terms have become common for compounds containing both one or more metal atoms and organic fragments: organometallic compounds and metal–organic coordination compounds. The main difference is that organometallic compounds in accordance with the standards of IUPAC nomenclature must contain at least one direct metal–carbon (M-C) bond, while the term metal–organic can be used more broadly to include compounds—where the organic part is bonded to the metal via a heteroatom like oxygen or nitrogen—or to refer to a larger framework structure like a metal–organic framework (MOF) where there is no direct M-C chemical bond.

An up-to-date overview of the fundamentals, synthesis, and applications of organometallic compounds can be found in Ref. [1].

In Ref. [2], a brief analysis of the existing definitions of the term “coordination compound” has been carried out, and a definition that takes into account the features of this class of chemical compounds has been proposed. The existing systematic IUPAC nomenclature of coordination compounds, its possible use for constructing the names of metal complexes of different types, as well as difficulties associated with this process have been considered.

Metal–organic frameworks (MOFs) are made by linking inorganic and organic units by strong bonds (reticular synthesis), which creates strong bonds between inorganic and organic units. In Ref. [3], the comprehensive review of work on physicochemical properties and application of MOF was presented.

This Special Issue presents examples of contemporary works that demonstrate the results of studies of the structural, spectral, energetic, and other properties of representative organometallic compounds, classical metal–organic coordination complexes, and metal–organic frameworks.

Based on the state of this area of research, the following problems associated with the further development of this specific area of chemical science can be distinguished:

1. *Improving synthetic methods aimed at obtaining metal–organic coordination compounds*. Basically, existing synthetic methods for obtaining compounds of this type can, in principle, be reduced to one of two options. For the first option, there will be a direct interaction between those metal ions and organic compounds, which will ultimately form the internal coordination sphere of the future complex. For the second option, organic compounds that will further create an inner sphere together with metal ions will not initially be present in the reaction system, but they will appear in it as a result of the interaction between some simpler organic compounds from among the starting materials of the reaction.
2. *Improving methods of physical and chemical analysis and theoretical methods for calculating the physical and chemical characteristics of metal–organic coordination compounds.* It should be noted right away that although the situation with methods of physicochemical analysis in this area of chemistry is generally good, there are still points that clearly require additional research. First of all, this relates to the problem of experimental determination of the molecular structures of metal–organic coordination compounds, which is successfully solved only in cases where the analyzed substance can be formed in the form of separate single crystals. However, in comparison with an individual molecule, the resulting structure turns out to be radically distorted by the collective interaction in the crystal as well as the specificity of X-ray scattering (in general, it is not the position of the nuclei that is determined, but the position of the centers of gravity of the electron density). To determine the molecular structures of substances in the gas phase, the use of gas electron diffraction is required. However, using the latter method is associated with a number of difficulties, namely that not all substances are capable of passing into the gas phase congruently, and the presence of sample decomposition products in the scattering volume can lead to cardinal errors in the results of structural analysis. A way out of this situation was found in [4,5,6], which combined simultaneous electron diffraction and mass spectrometric experiments, thereby making it possible to continuously monitor the composition of the vapor under study throughout the diffraction experiment, as well as monitor the presence of volatile impurities and oligomers. However, another problem in using gas electron diffraction is the low resolution of this method, which does not allow one to distinguish between close internuclear distances. To a certain extent, the problem can be solved by using quantum chemical data on the difference in such distances in structural analysis. However, the different physical meaning of the structure obtained by electron diffraction (averaged over all electronic, vibrational, and rotational states populated under experimental conditions) and the structure from quantum chemical calculations (corresponding to the minimum on the potential energy surface and is not observed experimentally) greatly complicates the problem and requires taking into account specifics of intramolecular dynamics when interpreting a diffraction pattern. This approach makes it possible to level out the large-scale error of quantum chemical calculations, but the determined structure acquires the physical meaning of semi-experimental.

At the same time, there are a limited number of theoretical works in the literature devoted to quantum chemical calculations of the above-mentioned metal complexes using the DFT method, and even fewer using even more advanced quantum chemical methods. In any case, there are significantly fewer works than those devoted to the synthesis of these compounds. This is partly due to the complexity of the molecular structures of many of these compounds, which means that their calculation using the DFT method, even with the simplest functionals and basis sets, is often very labor-intensive and resource-intensive, making it difficult to implement for many chemical researchers (although, undoubtedly, as computer technology improves, the severity of this problem will gradually decrease). On the other hand, it is necessary to further improve the methods of quantum chemical calculations themselves as applied to these compounds.

3. *Search for new ways and possibilities for practical application of metal–organic coordination compounds.* At a given moment in time, a number of options for using these compounds have already been tested to one degree or another—from catalysis to microelectronics, from optics and spectroscopy to medicine and pharmacology, from components of non-fading varnishes and paints to components of sensor systems, etc.—as “smart materials”. However, there are good reasons to believe that the applications for these compounds are by no means completely exhausted, and with the emergence and development of new, previously unknown branches of science and technology (for example, nano- and digital technologies), the scope of their application will be significantly expanded.

## 2. An Overview of the Published Articles

Contribution 1. The review by Gabriela Camarillo-Martínez and co-authors is devoted to the analysis of a new class of compounds: metal–organic frameworks. MOFs are hybrid materials that combine inorganic and organic components, characterized by their porous, crystalline, and network-like structures. These frameworks are constructed through the coordination of metal ions or clusters with organic ligands. These coordination polymers have repeating entities that extend in one, two, or three dimensions through various metal–ligand covalent bonds. The structural diversity of MOFs allows for the fine-tuning of properties like pore size, stability, and functionality, making them ideal for a wide range of industrial, environmental, and biomedical applications. Some of the most commonly used synthesis methods for the production of MOFs, as well as some reaction conditions, yield, and particle size, were systematized.

For example, this review considers the MOF Basolite C-300, [Cu_3_(btc)_2_(H_2_O)_3_]. The results of studies using different spectroscopic methods are considered: FTIR spectra displaying characteristic vibrational modes, Raman spectra showing vibrational features, UV–Vis depicting electronic properties, EPR spectra highlighting magnetic properties, NMR spectra identifying chemical environments, adsorption experiments (BET analysis), TGA and DSC analysis, electrochemical impedance spectroscopy EIS evaluating the electrochemical properties, different kinds of X-ray-based spectroscopy enabling surface-sensitive analysis, XRD structure analysis. Applications of MOFs and Basolite C-300 are also discussed.

Contribution 2. The article by Denis V. Chachkov and co-authors exemplifies the rapidly developing theoretical approach to searching for new macroheterocyclic compounds using quantum chemical calculations.

In 1972, the first paper focusing on a new type of macroheterocyclic compound based on isoindole–boron subphthalocyanine, with fluorine or chlorine atoms as extra ligands, was published [7]. To date, several dozen papers have been published on the synthesis and study of the diverse properties of subphthalocyanines. The overwhelming majority of these papers describe boron-containing complexes, supporting the established view that boron(III) remains a unique and virtually exclusive complexing agent for subphthalocyanines. Aluminum(III) and Ga(III) subphthalocyanines are also known, but they are much less stable, and relatively few studies have been devoted to them.

The article by Denis V. Chachkov and co-authors aims to search for similar compounds. In this article, using beryllium complexes with subporphyrazine and its mono-, di-, and tri[benzo]-annelated derivatives as examples, it is demonstrated that beryllium(II) can act as a complexing agent in submacroheterocyclic compounds. The beryllium complexes have a cone-shaped form characteristic of boron subphthalocyanines but do not contain extra ligands. Calculations were performed at the DFT/B3PW91/TZVP and DFT/M062X/def2-TZVP theoretical levels. The structure and standard thermodynamic parameters of formation (Δ_f_H°, S°, and Δ_f_G°) of the above-mentioned compounds were calculated, and the possibility of their existence was demonstrated.

Contribution 3. The article of Julio Corredoira-Vázquez and co-authors reports the synthesis of the 18-membered macrocycles 3,6,10,13-tetraaza-1,8(2,6)-dipyridinacyclotetradecaphane and 3,10-dimethyl-3,6,10,13-tetraaza-1,8(2,6)-dipyridinacyclotetradecaphane, L and L^Me2^, which contain ethylene and methylethylene spacers between their N_3_ moieties, and the use of both for the synthesis of two new dysprosium complexes: [DyL(OAc)_2_]OAc·7H_2_O·EtOH and [DyL^Me2^(Cl)_2_]Cl·2H_2_O.

Crystal structures of [DyL(OAc)_2_]OAc·7H_2_O·EtOH and [DyL^Me2^(Cl)_2_]Cl·2H_2_O were solved by X-ray diffraction. The disposition of the ligands is similar to that found in Cambridge Structural Database for the cations [SmL^Me2^(NO_3_)_2_]^+^, [CeL^Me2^(NO_3_)_2_]^+^, [DyL^cyh2^(NO_3_)_2_]^+^, and [YbL^cyh2^(NO_3_)_2_]. The crystal structures of the Dy^III^ complexes were compared with those of the lanthanoid and d-metal complexes of other 18-membered N_6_ donor macrocycles. Luminescent properties of [DyL(OAc)_2_]OAc·7H_2_O·EtOH and [DyL^Me2^(Cl)_2_]Cl·2H_2_O were also investigated experimentally, showing that both complexes are fluorescent, with the emission being sensitized by the ligands. Crystal structure of L·12H_2_O was solved and compared with those of other related macrocycles known in the literature.

Contribution 4. Yan-Xia Jin and Jin-Chang Guo developed the idea of the existence of planar tetra-, penta-, and hexacoordinated carbon compounds, extending it to planar complexes of other carbon subgroup elements—Si, Ge, Sn, and Pb—tetracoordinated by two boron atoms and two bismuth atoms. Using PBE0/def2-SVP calculations, the authors searched for possible stable structures of the XB_2_Bi_2_ type, where X = Si, Ge, Sn, and Pb. Five low-lying isomer structures were then re-optimized, and frequency analyses were performed at the PBE0-D3(BJ)/def2-TZVP theory level. The higher-level single-point calculations at CCSD(T)/def2-TZVP//PBE0-D3(BJ)/def2-TZVP were then carried out to obtain an accurate stability ordering of these five isomers.

The nature of chemical bonds in the energetically most stable complexes is discussed, while the dependence of HOMO and LUMO energies on the type of atom X is analyzed. Canonical Molecular Orbital (CMO) and Adaptive Natural Density Partitioning (AdNDP) analyses were performed. According to the CMO and AdNDP analyses, the ptSi/Ge/Sn/Pb clusters are governed explicitly by double 2π/2σ aromaticity.

To facilitate further experimental characterization, the IR spectrum of the ptX XB_2_Bi_2_ clusters was simulated at the theoretical level of PBE0-D3(BJ)/def2-TZVP.

Contribution 5. The article of Dmitriy Semyonov and co-authors reports the details of synthesis of a series of C- and B-substituted nido-carborane derivatives with a pendant pyridyl group, which further were used in synthesis as ligands in the complexation with [(Ph_3_P)_2_NiCl_2_] and [(Ph_3_P)_2_PdCl_2_] of six new metallacomplexes possessing unusual η^5^:κ^1^(*N*)-coordination of the metal center. The prepared compounds were characterized by using methods of ^1^H, ^13^C, ^11^B, and ^31^P NMR spectroscopy, as well as IR and UV spectroscopy and high-resolution ESI mass spectrometry. Using X-ray diffraction, the single crystal structures of 1-(NC_5_H_4_-2′-S)-1,2-C_2_B_10_H_11_, 1-(NC_5_H_4_-2′-CH_2_S)-1,2-C_2_B_10_H_11_, Cs[7-(NC_5_H_4_-2′-CH_2_S)-7,8-C_2_B_9_H_11_] *closo*- and *nido*-carboranes, as well as 3-Ph_3_P-3-(4(7)-NC_5_H_4_-2′-S)-closo-3,1,2-NiC_2_B_9_H_10_ and 3-Ph_3_P-3-(4(7)-NC_5_H_4_-2′-CH_2_S)-*closo*-3,1,2-NiC_2_B_9_H_10_ metallacarboranes, were determined.

When discussing the design of dicarbollide ligands with a pyridine group as a side group, the authors focus the readers’ attention on two important points. The first of these is the presence and length of a spacer between the carborane cage and the pyridyl ring. This determines the size and stability of the metallocycle formed during coordination. Clearly, in the absence of such a spacer, the formation of only a strained four-membered metallocycle is possible for most d-metals. The second point is the position of the substitution in the nido-carborane basket. Unlike the cyclopentadienide ligand, in which all the carbon atoms in the five-membered ring are identical, the pentagonal face of the dicarbollide ligand is formed by two carbon atoms and three boron atoms. Another less obvious point that can affect the stability and reactivity of metal complexes is the mutual orientation of the ligands.

Contribution 6. The nature of organic linker substrates plays an important role in gas sorption and separation, as well as in catalytic applications of metal–organic frameworks. The article by Viktoriia Torbina and co-authors is devoted to the study of the properties of the UiO-66 MOF built from Zr_6_O_4_(OH)_4_(CO_2_)_12_ clusters, which are distinguished by exceptional stability. Using a combination of IR spectroscopy and DFT calculations, the authors showed the influence of the organic linker nature on the efficiency of various acidic sites in the UiO-66 structure. The calculations conformed the lower and higher Lewis acidity of Zr coordinatively unsaturated sites on linker defects in the UiO-66 structure featuring electron-withdrawing and electron-donating groups in the therephthalate moiety, respectively. A model is proposed that allows us to differentiate various active sites on UiO-66 and clarify their behavior. The model can be used in the design and fine-tuning of the UiO-66 features for a wide range of applications.

Contribution 7. In the article by Sławomir Grabowski, using the concept of the triel trivalent centers possess six electrons, DFT calculations were applied to analyze the nature of intermolecular interactions in a number of crystal structures containing compounds in which a trivalent gallium center interacting with a carbon system acts as a Lewis base. In such structures, the intermolecular forces do not change the stable trigonal configurations of gallium center, particularly into the tetrahedral ones. The reason for this feature was analyzed using quantum chemistry techniques. NBO approach was applied to calculate the atomic charges, the energies of orbital–orbital interactions, Wiberg bond indices. QTAIM calculations were used to analyze characteristics of the bond critical points. The energy decomposition analysis, EDA, was used for the estimation of energy quasi-classical electrostatic interaction between the unperturbed charge distributions of atoms, the energy of charge transfer and polarization phenomena, the energy corresponding to electron charge shifts resulting from the complex formation, the energy of dispersion forces, as well as the energy of Pauli repulsion. Most crystal structures analyzed are characterized by the parallel (or near-parallel) arrangements of molecules containing the gallium centers; they may be classified as stacking-like arrangements.

Contribution 8. The article by Mihaela Birdeanu and co-authors addresses a specific problem: finding an effective sorbent for wastewater treatment to remove methyl red dye. Various formulations consisting of tetraethyl orthosilicate (TEOS), 3-aminopropyltrimethoxysilane (APTMOS), substituted porphyrin (TAPP), and Eu^3+^ dopant were tested. The study utilized a wide range of physical research methods: SEM + AFM + UV-VIS-NIR + FT-IR + sorption experiments (BET analysis). BET data show that TEOS-based silica materials provide optimal conditions as adsorbents with very high specific surface areas. In comparison, silica materials also containing APTMOS have a fourfold lower specific surface area and reduced sorption capacity. Two different sorption mechanisms of methyl red have been reported: one is based on surface adsorption in the case of TEOS-based materials, and the other involves sorption of the dye into pores in the case of APTMOS-containing silica materials.

## 3. Conclusions

Despite the fact that there are currently many publications indicating very significant progress in solving each of the three problems listed in the Introduction section, many questions still remain, and to claim that its potential has already been fully revealed would, in our opinion, be clearly premature. This Special Issue of the *International Journal of Molecular Sciences*, with the title indicated in the title of the given article, is intended to further develop and improve on the related scientific field. We would like to hope that this paper will make at least a small contribution to this specific yet important branch of modern chemistry, as well as to the emergence of further research in this direction.
